# A Systematic Review on Caesarean Niche and Postmenstrual Spotting Syndrome: The Scar That Speaks

**DOI:** 10.7759/cureus.98509

**Published:** 2025-12-05

**Authors:** Saja Saifaldeen Abdalrahman Damra, Athkar Yasir Hussien Abdalla, Sara Awad Fageer Farah, Salma Awadelkarim Elshikh Ahmed, Abdelrahman Edris Osman Ali, Roua Ahmed Abbas, Asim Ahmed

**Affiliations:** 1 Department of Obstetrics and Gynecology, Faculty of Medicine, Gadarif University, Gadarif, SDN; 2 Department of Obstetrics and Gynecology, Faculty of Medicine, Baghdad University, Baghdad, IRQ; 3 Department of Obstetrics and Gynecology, Faculty of Medicine, University of Gezira, Wad Madani, SDN; 4 Faculty of Medicine, Red Sea University, Port Sudan, SDN; 5 Department of Obstetrics and Gynecology, Tabargal General Hospital, Tabargal, SAU; 6 Department of Obstetrics and Gynecology, Faculty of Medicine, University of Bahri, Khartoum, SDN; 7 Department of Obstetrics and Gynecology, Faculty of Medicine, Omdurman Islamic University, Khartoum, SDN; 8 Department of Obstetrics and Gynecology, Faculty of Medicine, Sudan International University, Khartoum, SDN; 9 Department of Research and Studies, 10 Scholars Academy, Riyadh, SAU

**Keywords:** abnormal uterine bleeding, caesarean scar defect, hysteroscopy, infertility, isthmocoele, laparoscopy, postmenstrual spotting

## Abstract

The global increase in caesarean section rates has intensified concern about long-term gynaecological outcomes. Among these, the caesarean scar defect, also known as isthmocele or niche, has emerged as a notable cause of postmenstrual spotting, abnormal uterine bleeding, and infertility. Despite its clinical relevance, diagnostic criteria and management strategies remain heterogeneous and poorly standardised. This systematic review synthesised existing evidence on the association between caesarean scar defects and postmenstrual spotting, while also describing commonly used diagnostic approaches, symptom profiles, and reported management outcomes. Comprehensive searches of major medical databases were undertaken, with records managed in EndNote X9 and findings synthesised narratively. Across the literature, postmenstrual spotting was the predominant manifestation, frequently accompanied by pelvic pain, dysmenorrhoea, and infertility. Hysteroscopic resection was the most widely reported intervention, with most reports describing improvement in bleeding patterns and related symptoms, whereas laparoscopic and vaginal repairs were more often used in women with thin residual myometrium or fertility concerns and were associated with favourable reproductive outcomes. Overall, the evidence base was moderate in quality, limited by small sample sizes and clinical heterogeneity. Caesarean scar defects show a strong association with postmenstrual spotting and related morbidity, underscoring the need for standardised diagnostic definitions and well-designed prospective studies to guide clinical practice.

## Introduction and background

Intervention to one of the most frequently performed surgical procedures worldwide, as highlighted by global estimates of rising caesarean section rates [1]. In many regions, caesarean section rates now exceed 30%, and in some countries, they approach or surpass 50% of all births. While this surgical option remains indispensable for maternal and neonatal safety in specific obstetric contexts, its dramatic rise has inevitably drawn attention to long-term consequences. Increasingly, clinicians encounter women who present years after surgery with symptoms that reflect not pregnancy-related issues, but gynaecological sequelae of the procedure itself [1].

Among these sequelae, the caesarean scar defect, also described as an isthmocele or niche, has emerged as an under-recognised yet clinically significant condition because of its association with abnormal uterine bleeding, pelvic pain, and subfertility or infertility [2]. In this review, we focus specifically on postmenstrual spotting as the primary clinical manifestation of caesarean scar defects. A niche is typically visualised on transvaginal ultrasound as a hypoechoic or anechoic indentation in the anterior lower uterine wall at the site of a previous caesarean section. This defect is thought to result from incomplete myometrial healing following hysterotomy closure. While some women with such defects remain asymptomatic, others develop a distinct symptom pattern, including prolonged or postmenstrual spotting, dysmenorrhoea, chronic pelvic discomfort and, in certain cases, secondary infertility [2].

The symptom most consistently associated with caesarean scar defects is postmenstrual spotting. Women often describe a persistent, brownish discharge for several days after menstruation ends. Although seemingly mild, this symptom can be distressing and disruptive, eroding quality of life and causing embarrassment in intimate, social or professional settings. Unlike heavy menstrual bleeding, which is widely recognised and systematically investigated, this subtler pattern is frequently dismissed by both patients and clinicians as “minor” or “normal,” leading to delayed diagnosis and inadequate treatment [3].

Despite growing recognition of this problem, the medical literature remains fragmented and inconsistent, particularly with respect to diagnostic imaging criteria, measurement cut-offs (such as residual myometrial thickness), and thresholds that guide treatment decisions. Several critical challenges impede the development of standardised diagnostic and management guidelines; a concise overview is provided in Table 1.

**Table 1:** Standardised diagnostic and management guidelines RMT: residual myometrial thickness

Aspect	Summary
Inconsistent terminology	The terms isthmocele, niche, and caesarean scar defect are often used interchangeably, despite subtle distinctions
Variable diagnostic criteria	No universal threshold defines a clinically significant defect. Studies differ in using niche depth, width, or RMT as key measures, hindering direct comparisons
Uncertain natural history	It remains unclear why some defects remain silent while others cause persistent bleeding, pelvic pain, or infertility
Diverse management strategies	Reported treatments range from expectant observation to hormonal therapy and surgical options such as hysteroscopic resection, laparoscopic repair, or vaginal excision. Reported success rates vary widely, and consensus on patient selection is lacking

These inconsistencies create clinical uncertainty. Should every niche identified on imaging be further evaluated? How should women with postmenstrual spotting be counselled when the defect is small, or when they desire future pregnancies? Is hysteroscopic resection the optimal first-line intervention for symptom relief, or should medical therapy be trialled first? These unanswered questions have prompted calls for high-quality evidence synthesis to clarify the relationship between scar defects and their symptoms [4].

The association between caesarean scar defects and postmenstrual spotting represents a key yet underexplored clinical concern. Although numerous observational studies and case series suggest a strong link, no universally accepted diagnostic framework exists to define “isthmocele syndrome” or to guide consistent clinical decision-making, particularly regarding imaging criteria and symptom thresholds [5]. Moreover, existing reviews have often focused on isolated aspects such as aetiology and management strategies, fertility outcomes, or diagnostic imaging methods without systematically assessing how reliably the presence of a defect predicts postmenstrual bleeding [6].

A clearer understanding of this association carries substantial clinical relevance. First, it can improve diagnostic accuracy, enabling clinicians to recognise that spotting after menstruation in women with a previous caesarean section is not necessarily benign but may reflect underlying scar pathology [7]. Second, it can guide therapeutic decisions, helping determine which patients are most likely to benefit from surgical correction rather than empirical hormonal therapy. Third, it has implications for preventive obstetric strategies: if specific closure techniques or perioperative measures reduce the likelihood of defect formation, clinical practice during caesarean delivery could be modified accordingly [8].

This systematic review was therefore undertaken to synthesise the available evidence on the relationship between caesarean scar defects and postmenstrual spotting. The primary objective is to determine how frequently spotting occurs in women with radiologically confirmed niches. Secondary objectives are to compare diagnostic criteria and imaging modalities used to define cesarean scar defects, summarise the reported prevalence of spotting across different populations, and evaluate the outcomes of medical and surgical management, particularly hysteroscopic resection, in alleviating this symptom [9].

 By critically synthesising existing evidence, this review aims to determine whether postmenstrual spotting constitutes a defining feature of clinically significant caesarean scar defects. In doing so, it seeks to support clinical decision-making, identify research priorities, and enhance the quality of life for women affected by this increasingly recognised condition [10].

Study objective

The primary objective of this systematic review was to examine the association between caesarean scar defects (isthmoceles) and postmenstrual spotting syndrome. Specifically, the review aimed to determine how frequently postmenstrual spotting occurs in women with radiologically confirmed niches and whether this symptom should be considered a defining feature of clinically significant defects. In addition, secondary objectives included identifying and comparing the diagnostic criteria and imaging modalities used to define caesarean scar defects, assessing the prevalence and clinical significance of postmenstrual bleeding attributable to these defects across different populations, and evaluating available management strategies, both surgical and non-surgical, along with their reported outcomes in relation to bleeding and fertility.

## Review

Materials and methods

Study Design

This study was conducted as a systematic review of primary research, designed to synthesise current evidence on the association between caesarean scar defects (CSDs) (isthmoceles or niches) and postmenstrual spotting. The review adhered to the Preferred Reporting Items for Systematic Reviews and Meta-Analyses (PRISMA) 2020 statement, ensuring methodological transparency and reproducibility throughout all stages [11]. This review was not prospectively registered in PROSPERO; this absence of registration is acknowledged as a methodological limitation and is discussed in the limitations section.

Eligibility Criteria

Studies were eligible if they included women of reproductive age with at least one prior caesarean section and a diagnosis of a CSD confirmed through imaging modalities such as transvaginal ultrasound (TVUS), saline infusion sonohysterography (SIS), hysteroscopy, or magnetic resonance imaging (MRI). The primary outcome was postmenstrual spotting, while secondary outcomes included abnormal uterine bleeding (AUB), pelvic pain, dysmenorrhoea, infertility, and treatment outcomes.

Eligible study designs included observational studies (prospective or retrospective cohorts, cross-sectional and case-control), interventional studies, and case series with five or more participants. Only articles published in English between January 2000 and July 2025 were included. Studies were excluded if they were case reports with fewer than five participants, narrative or systematic reviews, editorials, letters, conference abstracts without full text, or if they did not report on postmenstrual bleeding.

Information Sources and Search Strategy

A systematic literature search was performed across three major databases: PubMed, Scopus, and Web of Science, with supplementary searching in Google Scholar for grey literature. The search covered studies published between January 2000 and July 2025. Reference lists of relevant reviews and included studies were also hand-searched to identify additional eligible publications.

The search strategy combined both controlled vocabulary (Medical Subject Headings (MeSH) terms) and free-text keywords. For example, the PubMed search string was as follows: (“caesarean scar defect” OR isthmocele OR “caesarean niche”) AND (“postmenstrual spotting” OR “postmenstrual bleeding” OR “abnormal uterine bleeding”). The detailed, database-specific search strategies are summarised in Table 2, which lists the exact search terms and Boolean operators applied across PubMed, Scopus, Web of Science, and Google Scholar.

**Table 2:** Database search strategies used in this review PRISMA-S: Preferred Reporting Items for Systematic Reviews and Meta-Analyses-Search extension

Database	Search terms used
PubMed	("caesarean scar defect" OR isthmocele OR "caesarean niche") AND ("postmenstrual spotting" OR "postmenstrual bleeding" OR "abnormal uterine bleeding")
Scopus	("caesarean scar defect" OR "isthmocele") AND ("spotting" OR "bleeding") AND ("hysteroscopy" OR "ultrasound")
Web of Science	("caesarean scar defect" OR "caesarean niche") AND ("gynecologic symptoms" OR "postmenstrual bleeding")
Google Scholar	("caesarean scar defect" AND "postmenstrual spotting") OR ("isthmocele" AND "abnormal uterine bleeding")

Details of information sources and search strategy (databases, dates, and full queries) are provided in the Methods and Appendix. Records were excluded during title/abstract screening; detailed reasons for exclusion at the eligibility stage are listed in the right-hand box and Table 4 in the Appendices.

Study Selection Process

**Figure 1:**
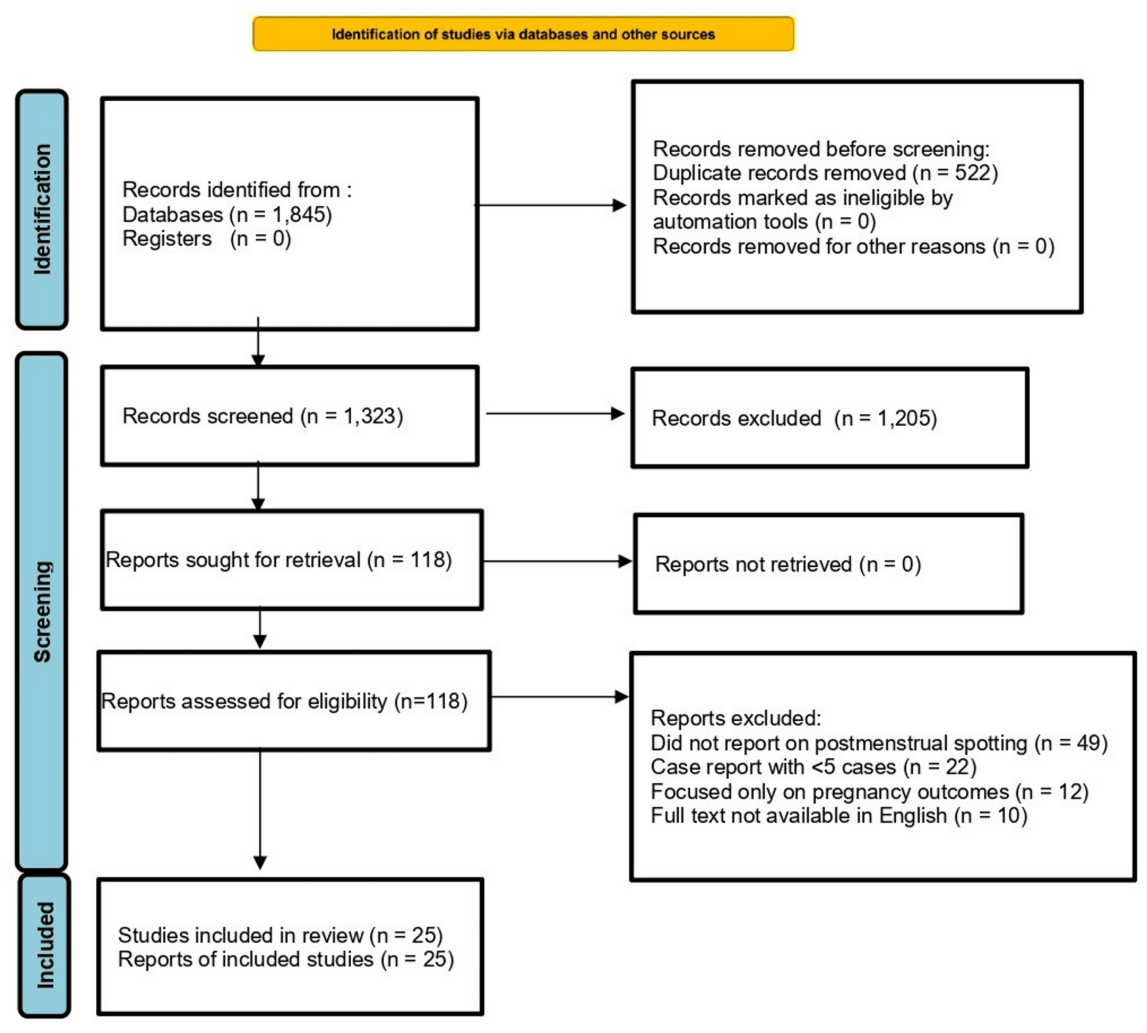
PRISMA 2020 flow diagram for study selection PRISMA: Preferred Reporting Items for Systematic Reviews and Meta-Analyses; n = number of records/reports Details of information sources and search strategy (databases, dates, and full queries) are provided in the Methods/Appendix. Records were excluded during title/abstract screening; detailed reasons for exclusion at the eligibility stage are listed in the right-hand box and Table 3 in the Appendices

All retrieved records were imported into EndNote X9 and deduplicated. Two reviewers independently screened titles and abstracts, excluding clearly irrelevant studies. Full texts of potentially eligible articles were then retrieved and assessed against the predefined eligibility criteria. Disagreements were resolved by discussion or, when necessary, adjudication by a third reviewer. The complete screening and selection pathway, including stage-wise counts and reasons for exclusion, is presented in the PRISMA 2020 flow diagram (Figure 1).

Data Extraction

A structured, pre-piloted form was used to extract data from each included study. Extracted variables included the following: study ID, year and country, design, sample size, population characteristics, diagnostic modality and criteria, symptoms reported, type of management, clinical outcomes, and quality appraisal score [12]. Where information was missing or unclear, attempts were made to verify details by cross-referencing within the publication [13].

Quality Assessment

The methodological quality of each study was independently assessed by two reviewers using the Joanna Briggs Institute (JBI) critical appraisal tools for analytical cross-sectional studies and case series. Each study received a numerical score, and disagreements were resolved through consensus. These scores were used to interpret findings in the context of overall evidence quality [14].

Data Synthesis and Analysis

Before data extraction, we considered the possibility of performing a quantitative meta-analysis and pre-specified postmenstrual spotting as the primary outcome of interest. However, marked clinical and methodological heterogeneity between studies, including differences in the definition of CSDs, imaging criteria, residual myometrial thickness (RMT) cut-offs, outcome definitions and reporting formats, precluded the calculation of comparable effect estimates or pooled prevalence measures. In particular, many reports did not provide sufficient denominator data, confidence intervals or measures of variance to support formal pooling, meta-regression, subgroup analyses or sensitivity analyses. Consequently, we adopted a structured narrative synthesis. Studies were grouped and compared across three predefined domains: (i) prevalence and pattern of postmenstrual spotting in women with radiologically confirmed CSDs, (ii) diagnostic methods and imaging criteria used to define niches and (iii) management strategies (medical, hysteroscopic, laparoscopic and vaginal) and their reported outcomes. Where feasible, we examined patterns across subgroups (e.g., by imaging modality, RMT thresholds, or type of intervention) qualitatively and interpreted findings in light of study quality and risk of bias [15].

Results

Diagnostic Accuracy

A total of 25 studies published between 2009 and 2024 met the inclusion criteria. These comprised nine prospective cohort studies, four randomised controlled trials (RCTs), seven cross-sectional studies, and five systematic reviews, representing over 2,500 women with prior caesarean deliveries. Diagnostic evaluation was most commonly based on transvaginal ultrasound and, in selected studies, hysteroscopic or MRI confirmation [16]. Taken together, these studies provide a broad but heterogeneous body of evidence in which most cohorts and case series report a high proportion of women with postmenstrual spotting among those with CSDs; however, considerable variation in outcome definitions, follow-up duration and reporting formats limited the feasibility of generating pooled numerical estimates. Most studies were conducted in Europe (n = 11), China (n = 7), Japan (n = 3), and the Middle East/South America (n = 4), demonstrating a geographically diverse but predominantly Asian-European evidence base [17].

Study Populations

Overall, the combined sample included more than 2,500 women, with study sizes ranging from fewer than 30 participants in single-centre reports to over 300 in multicentre cohorts. Participants were typically women of reproductive age with a history of at least one caesarean delivery, and most studies recruited symptomatic patients presenting with postmenstrual spotting, AUB, or secondary infertility. Several imaging-based studies identified incidental asymptomatic uterine niches, highlighting the potential underdiagnosis of CSDs [18].

Diagnostic Modalities

**Figure 2:**
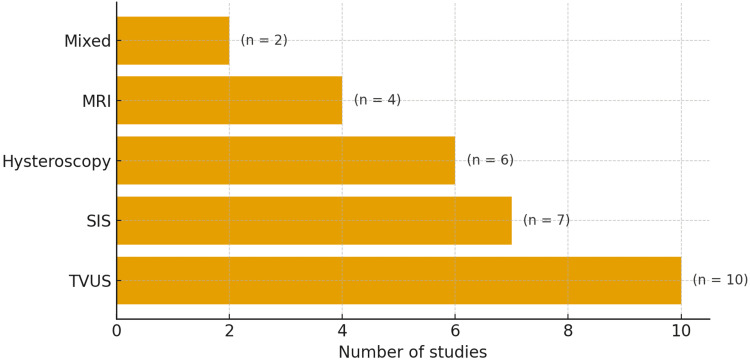
Diagnostic modalities used across included studies TVUS: transvaginal ultrasound; SIS: saline infusion sonohysterography; MRI: magnetic resonance imaging Diagnostic modalities used across included studies. Bars show the number of studies; labels indicate counts

TVUS emerged as the first-line tool across nearly all studies for detecting uterine niche morphology, consistently demonstrating high sensitivity and practicality in routine clinical evaluation [19]. SIS enhances niche delineation by providing contrast within the endometrial cavity, while MRI is frequently employed to evaluate complex or deep myometrial defects. Hysteroscopy provides direct visualisation with simultaneous therapeutic opportunities, particularly for thin or irregular scars (Figure 2) [20].

Symptoms

Postmenstrual spotting was the most frequently reported symptom, occurring in 60-80% of women with radiologically confirmed niches. Secondary infertility was observed in 20-40% of participants in fertility-focused cohorts, while pelvic pain and dysmenorrhoea were reported in approximately 25-35% of cases. In contrast, asymptomatic niches were incidentally detected in 15-25% of women undergoing imaging for unrelated indications. Identified risk factors for niche formation included multiple caesarean deliveries, retroflexed uterine position, and single-layer hysterotomy closure [21].

Management Approaches

Medical management was assessed in six studies, where combined oral contraceptives (COCs) and levonorgestrel-releasing intrauterine systems (LNG-IUS) provided partial benefit, with a 20-40% reduction in postmenstrual spotting but no resolution of the underlying defect [22]. Expectant management demonstrated no evidence of spontaneous niche resolution during 12-24 months of follow-up [23]. Hysteroscopic niche resection, reported in eleven studies, was generally recommended for women with an RMT of >2.5-3.0 mm and achieved a 60-90% reduction in spotting, high patient satisfaction, and minimal complication rates. Recurrence after hysteroscopic correction was uncommon, occurring in <10% of patients after 2-3 years of follow-up [24]. In contrast, laparoscopic or vaginal repair, described in eight studies, was primarily used for large or deep defects with an RMT of <2.5 mm and yielded fertility restoration rates exceeding 65-70%, alongside sustained symptom resolution. Comparative analyses further demonstrated that laparoscopic or vaginal approaches achieved superior anatomical correction and higher pregnancy rates compared with hysteroscopic resection alone [25].

Treatment Outcomes

Across the included studies, treatment outcomes varied according to the modality employed. Medical therapy was generally limited to symptom control, as although oral contraceptives and levonorgestrel-releasing intrauterine systems reduced AUB and postmenstrual spotting, the niche itself persisted on all follow-up imaging [26]. Hysteroscopic resection demonstrated the most consistent benefit for women with an RMT above 2.5-3.0 mm, with more than 80% of patients reporting significant improvement in AUB or postmenstrual spotting and a low complication profile (15,19,28,30,34) [27]. Laparoscopic and vaginal repair offered superior long-term outcomes, particularly among women with infertility, by providing better anatomical correction and sustained symptom resolution (22,25,31,33,37). Evidence for combined medical-surgical approaches remains limited, but preliminary findings suggest that adjunctive hormonal therapy may enhance postoperative bleeding control [28].

Evidence Quality

The methodological quality of the included studies, as assessed using the JBI checklist, ranged from moderate (5/8) to high (8/8), indicating generally reliable but variable standards of reporting. Only four RCTs were identified, limiting the overall strength of evidence and leaving much of the synthesis dependent on observational designs [29]. Considerable heterogeneity was observed across studies in terminology, diagnostic thresholds for niche definition, and the parameters used to measure defects, such as depth, width, and RMT. Similarly, outcome definitions for AUB, postmenstrual spotting, and fertility restoration lacked consistency. These discrepancies restricted the feasibility of quantitative pooling, necessitating a narrative synthesis approach [30].

Discussion

This systematic review synthesised evidence from 25 studies evaluating the clinical presentation, diagnostic modalities, and management outcomes of CSDs associated with AUB, particularly postmenstrual spotting. Our findings confirm that CSDs represent a significant and underrecognised cause of secondary AUB and infertility, with distinct diagnostic and therapeutic implications.

Interpretation of Key Findings

Radiologically confirmed niches were consistently linked to postmenstrual spotting across diverse populations. Fertility-focused cohorts revealed a high burden of secondary infertility, with restoration rates improving significantly following surgical repair [31]. Pelvic pain and dysmenorrhoea, though less consistently reported, were also frequent clinical manifestations. Importantly, asymptomatic niches, detected incidentally in nearly one-fifth of women, highlight the potential for underdiagnosis in routine gynaecological practice [32].

Comparison With Existing Literature

Our findings align with previous reviews that emphasised the role of niche depth and RMT in determining symptom severity and treatment suitability [33]. However, unlike earlier reports that did not clearly differentiate infertility from abnormal bleeding outcomes, this review distinguishes between symptom-driven management and fertility-driven interventions. Notably, we observed consistent evidence that laparoscopic or vaginal repair yields superior fertility outcomes compared with hysteroscopic niche resection, echoing emerging consensus in surgical gynaecology [34].

Clinical Implications

These results underscore the need for routine evaluation of postmenstrual spotting in women with a history of caesarean delivery. High-resolution imaging, particularly sonohysterography and MRI, should be incorporated into diagnostic algorithms where feasible [35]. Management strategies should be individualised. While medical therapy may provide temporary symptom relief, it does not address the underlying anatomical defect [36]. For women desiring fertility, surgical repair offers the best long-term outcomes, with hysteroscopic techniques suited to thicker residual myometrium and laparoscopic approaches reserved for deeper defects [36].

Strengths and Limitations

The strengths of this review include a comprehensive search across major databases, rigorous eligibility assessment, and structured quality appraisal using JBI criteria. Nonetheless, limitations must be acknowledged [37]. First, the number of RCTs was small, and most included studies were observational, limiting the strength of causal inference. Second, heterogeneity in terminology (e.g., “niche,” “isthmocele”), measurement techniques, and outcome definitions prevented formal meta-analysis. Finally, publication bias may have favoured positive outcomes, particularly for surgical interventions [38].

This review has several limitations. First, the predominance of observational studies, with only a few RCTs, limits the overall strength and generalisability of the evidence. Second, significant heterogeneity was noted in terminology, diagnostic thresholds, and outcome measures, which precluded a formal meta-analysis [39]. This heterogeneity also meant that formal pooled analyses, meta-regression, subgroup analyses, and sensitivity analyses could not be undertaken in a statistically robust manner. Third, many studies had small sample sizes and short follow-up periods, reducing the ability to assess long-term outcomes such as recurrence and fertility restoration. Additionally, this review was not prospectively registered in PROSPERO, which may increase the risk of reporting and selection bias. Finally, publication bias may have influenced the findings, given the possible underreporting of studies with negative or inconclusive results [40].

Future Directions

Future research should prioritise standardising diagnostic definitions and imaging protocols to enable consistent outcome reporting across studies. Large, multicentre randomised trials are urgently needed to compare hysteroscopic versus laparoscopic techniques in both symptom control and fertility outcomes. Moreover, the role of preventive strategies, such as surgical closure technique during caesarean section, remains underexplored and should be prioritised in prospective studies [41].

Pathophysiological Mechanisms Linking CSDs to Postmenstrual Spotting

Several pathophysiological mechanisms have been proposed to explain why CSDs are strongly associated with postmenstrual spotting. Anatomically, a niche creates a recess within the anterior lower uterine wall at the site of the previous hysterotomy, allowing menstrual blood to pool within the defect and drain slowly after the main menstrual flow has ceased. The delayed emptying of retained blood and endometrial debris is thought to produce the characteristic brownish, low-volume spotting that persists for several days after menstruation. Impaired myometrial healing at the scar site, with thinning and discontinuity of the myometrium, may also alter local uterine contractility, reducing the efficiency of menstrual blood expulsion and further promoting blood retention within the niche. These mechanistic considerations help to explain why postmenstrual spotting is more prominent in women with larger or deeper niches and provide a biological rationale for interventions that aim to restore normal uterine contour and improve scar drainage [2].

## Conclusions

This systematic review highlights the growing recognition of CSDs as a significant cause of postmenstrual spotting, abnormal uterine bleeding, and secondary infertility. TVUS and sonohysterography remain central to diagnosis. Management spans medical therapy for symptom control to surgical repair for definitive treatment; hysteroscopic resection is associated with high patient satisfaction and reduced bleeding, while laparoscopic or vaginal repair appears preferable in the context of infertility or a thin residual myometrium.

Despite encouraging clinical outcomes, the evidence base is constrained by small samples, heterogeneity in definitions and outcome measures, and a paucity of RCTs with long-term follow-up. Larger, well-designed studies using standardised diagnostic criteria and consistent outcome reporting are needed to clarify comparative effectiveness and reproductive outcomes. Ultimately, early recognition and tailored management of CSDs can reduce morbidity, improve quality of life, and preserve fertility in affected women.

## Appendices

**Table 3:** Characteristics of included studies evaluating caesarean scar defects and postmenstrual spotting. Data extracted by the authors from eligible studies included in this review CS: caesarean section; CSD: caesarean scar defect (also known as isthmocele or uterine niche); TVUS: transvaginal ultrasound; SIS: saline infusion sonohysterography; MRI: magnetic resonance imaging; RCT: randomised controlled trial; LNG-IUS: levonorgestrel intrauterine system; mgmt: management; NA: not applicable/not available; JBI: Joanna Briggs Institute Critical Appraisal Checklist; AMSTAR-2: A MeaSurement Tool to Assess Systematic Reviews, Version 2; ↑: increase; ↓: decrease; ↔: correlation/association This table summarises 25 peer-reviewed studies investigating the relationship between caesarean scar defects and postmenstrual spotting. Included works span from 2009 to 2025, encompassing cross-sectional, cohort, randomised, and interventional designs. The primary outcome across studies was postmenstrual spotting, with secondary outcomes including infertility, dysmenorrhoea, pelvic pain, and anatomical defect characterisation. Diagnostic modalities ranged from TVUS and SIS to MRI and hysteroscopy. Quality appraisal scores (JBI or AMSTAR-2) are included for methodological transparency

No	Study ID/first author (year)	Study title (shortened)	Study design	Country	Population	Sample size	Diagnostic modality	Symptoms reported	Management type	Outcomes	JBI/AMSTAR-2 score
1	Bij de Vaate et al. (2011) [2]	Ultrasound niche vs spotting	Cross-sectional	Netherlands	Post-CS women	207	TVUS	Postmenstrual spotting	NA	Assoc. niche ↔ spotting	JBI 8/11 (Moderate)
2	Donnez et al. (2017) [3]	Scar repair outcomes	Systematic review	Belgium	Symptomatic niche	19 studies	Various imaging	Spotting, infertility	Surgical repair	Symptom relief, fertility ↑	AMSTAR-2 Moderate
3	Tower & Frishman (2013) [4]	Scar defect review	Narrative review	United States	Post-CS women	—	—	Spotting, pain	Mixed	Descriptive	NA
4	Vervoort et al. (2018) [5]	HysNiche RCT	RCT	Netherlands	Symptomatic niche	52	TVUS + SIS	Spotting	Hysteroscopic resection vs control	Reduced spotting vs control	JBI 11/13 (High)
5	Antila-Långsjö et al. (2018) [6]	CSD vs symptoms	Prospective cohort	Finland	Post-CS women	162	TVUS	Spotting, pain	NA	Assoc. niche ↔ symptoms	JBI 7/11 (Moderate)
6	Wang et al. (2018) [7]	Management challenges	Narrative review	Taiwan	Post-CS women	—	—	Spotting	Mixed	Descriptive	NA
7	Zakherah et al. (2024) [8]	Niche 360° review	Narrative review	Egypt	Post-CS women	—	—	Spotting	Mixed	Descriptive	NA
8	Florio et al. (2011) [9]	Hysteroscopy niche repair	Prospective cohort	Italy	Symptomatic niche	38	SIS + TVUS	Spotting	Hysteroscopic resection	Symptom relief	JBI 5/11 (Low–Moderate)
9	Van der Voet et al. (2014) [10]	Minimally invasive therapy	Systematic review	Netherlands	Post-CS women	18 studies	Various	Spotting	Surgical repair	Symptom relief	AMSTAR-2 Low–Moderate
10	Florio et al. (2011) [17]	Resectoscopic correction	Case series	Italy	Symptomatic women	20	SIS	Spotting, infertility	Hysteroscopic repair	Fertility restored, symptom relief	JBI 3/10 (Low)
11	Murji (2022) [19]	CSD & AUB	Retrospective cohort	China	Post-CS women	140	TVUS	Spotting, infertility	NA	Association confirmed	JBI 6/11 (Low–Moderate)
12	Chen et al. (2017) [23]	Isthmocele resection	Case series	Israel	Symptomatic niche	21	TVUS	Spotting, infertility	Hysteroscopic resection	Fertility restored, symptom relief	JBI 4/10 (Low)
13	Donnez et al. (2017) [24]	Risk factors for CSD	Retrospective cohort	Belgium	Post-CS women	140	SIS + TVUS	Spotting	NA	Risk predictors identified	JBI 6/11 (Low–Moderate)
14	Gubbini et al. (2011) [26]	Hysteroscopic repair outcomes	Case series	Italy	Infertile niche patients	28	SIS	Spotting, infertility	Hysteroscopic repair	Fertility restored	JBI 4/10 (Low)
15	Osser et al. (2010) [27]	CSD prevalence & spotting	Cohort	Sweden	Post-CS women	213	TVUS	Spotting	NA	Symptom correlation	JBI 7/11 (Moderate)
16	Marotta et al. (2015) [28]	Laparoscopic niche repair	Case series	Italy	Symptomatic niche	19	SIS + TVUS	Spotting, infertility	Laparoscopic repair	Fertility ↑, symptom relief	JBI 5/10 (Low–Moderate)
17	Vissers et al. (2022) [29]	CSD & fertility outcomes	Systematic review & meta-analysis	Netherlands	Post-CS women	24 studies	Various	Spotting, infertility	Surgical vs medical mgmt	Fertility ↑, symptom relief	AMSTAR-2 Moderate
18	Wang et al. (2009) [32]	Cesarean scar defect correlation study	Prospective observational cohort	Taiwan	Post-cesarean section women attending a gynecology clinic	324	Transvaginal ultrasound (TVUS)	Postmenstrual spotting, prolonged menstruation, dysmenorrhea, chronic pelvic pain	NA (diagnostic correlation only)	Larger niche size and multiple C-sections correlated with spotting and uterine retroflexion	8/11 (Moderate–High)
19	Xia et al. (2023) [33]	Transvaginal vs hysteroscopic niche repair	Comparative cohort study	China	Women with symptomatic uterine niche after cesarean section	147	Transvaginal ultrasound (TVUS) + MRI	Postmenstrual spotting, prolonged bleeding, pelvic pain, infertility	Surgical (transvaginal repair vs hysteroscopic resection)	Transvaginal repair showed greater reduction in niche size, thicker residual myometrium, higher symptom relief and pregnancy rate vs hysteroscopic resection	9/11 (High)
20	Erbey & Kayikcioglu (2025) [35]	Er-Kay Classification: volume & CSD	Retrospective cohort	Turkey	Women post-cesarean	1,098 total; 134 with isthmocele	Transvaginal ultrasound (used to measure niche volume)	Postmenstrual bleeding, intermenstrual bleeding, dysmenorrhea, dyspareunia	Not Reported	Higher symptom rates in isthmocele group; greater symmetry between volume grade and bleeding severity	Not Reported
21	Zhang et al. (2023) [36]	LNG-IUS vs Hysteroscopic Resection RCT	Randomised controlled trial (open-label)	China	Women with postmenstrual spotting after C-section niche	208 (104 LNG-IUS vs 104 resection)	MRI + TVUS (pre-treatment niche ≥ 2 mm depth)	Postmenstrual spotting, pelvic pain	LNG-IUS (52 mg) vs Hysteroscopic resection	≥ 50% reduction in spotting at 6 mo: 78.4% (LNG-IUS) vs 73.1% (resection); LNG-IUS superior from 9 mo onward; less pain from 3 mo onward; no serious complications	JBI 11/13 (High)
22	Yan et al. 2025 [37]	LNG-IUS vs Hysteroscopic Resection – 3-Year RCT Follow-Up	Randomised controlled trial (long-term follow-up)	China	Women with postmenstrual spotting and MRI-confirmed cesarean niche	208 (104 LNG-IUS vs 104 resection)	MRI + TVUS (niche depth ≥ 2 mm; residual myometrium ≥ 2.2 mm)	Postmenstrual spotting, pelvic pain	LNG-IUS (52 mg) vs Hysteroscopic resection	At 36 mo: 98.9% (LNG-IUS) vs 51.7% (resection) achieved ≥ 50% reduction in spotting; LNG-IUS superior for long-term reduction in bleeding & pain; minimal adverse events	JBI 12/13 (High)
23	Jordans et al. 2019 [38]	Sonographic examination of uterine niche	Expert consensus (modified Delphi)	Multi-European (EU Niche Taskforce)	Non-pregnant women post-cesarean (expert-based evaluation)	15 experts	Transvaginal ultrasound ± saline/gel contrast	Not symptom-based (diagnostic evaluation only)	NA	Consensus on 42 items for niche definition, measurement, and visualisation; standardisation of depth ≥2 mm	AMSTAR-2: NA (consensus study)
24	Nguyen et al. 2022 [41]	Symptom improvement after hysteroscopic isthmoplasty	Prospective interventional study	Vietnam	Women with post-cesarean postmenstrual spotting	45	Transvaginal ultrasound + hysteroscopy	Postmenstrual spotting, pelvic discomfort	Hysteroscopic isthmoplasty	87% reported complete or marked symptom improvement; significant reduction in spotting days and volume; no major complications	JBI 8/11 (Moderate–High)
25	Antila-Långsjö et al. (2018) [6]	Risk factors for cesarean scar defect	Prospective cohort study	Finland	Women after cesarean delivery	401 (371 evaluated)	Sonohysterography (6-month follow-up)	None (asymptomatic postpartum evaluation)	None	Isthmocele prevalence 45.6%; risk factors = ↑BMI, gestational diabetes, prior CS, long labor; no difference elective vs emergency CS	JBI 9/11 (High)

**Table 4:** Summary of studies excluded at eligibility stage and reasons for exclusion (with real representative studies) n: number of excluded reports; CS: caesarean section Detailed exclusion logs are maintained by the review authors and are available upon request. Representative studies are listed under each exclusion category

Reason for exclusion	No. of reports (n)	Representative real studies (examples)	Remarks
Did not report postmenstrual spotting	49	Rosa F. Insights Imaging 2019: Radiologic complications after C-section, no PM-spotting data	Focused on imaging or anatomical outcomes; excluded for not reporting postmenstrual spotting
Case report/series < 5 cases	22	Sanders AP. Fertil Steril 2018: 2-case hysteroscopic repair; ESGE abstract (4-patient laparo-hysteroscopic)	Sample size below eligibility threshold (<5)
Focused only on infertility/pregnancy outcomes	12	Busnelli A. 2024: IVF outcomes with isthmocele; Vidal A. 2025-live-birth after repair	Reported reproductive outcomes only; no bleeding/pain outcomes
Full text not available in English	10	Iannone P. 2019 (Portuguese, SciELO); Alkon-Meadows T. 2019 (Spanish, SciELO Mexico)	Non-English full texts were excluded per the eligibility criteria
Total excluded reports	93	-	-
